# Hospitals Bending the Cost Curve With Increased Quality: A Scoping Review Into Integrated Hospital Strategies

**DOI:** 10.34172/ijhpm.2021.168

**Published:** 2021-12-08

**Authors:** Erik Wackers, Niek Stadhouders, Anthony Heil, Gert Westert, Simone van Dulmen, Patrick Jeurissen

**Affiliations:** ^1^Radboud University Medical Center, Radboud Institute for Health Sciences, IQ Healthcare, Nijmegen, The Netherlands.; ^2^Ministry of Health, Welfare, and Sport, The Hague, The Netherlands.

**Keywords:** Scoping Review, Hospital Strategy, Quality Improvement, Cost Reduction, Implementation

## Abstract

**Background:** A lack of knowledge exists on real world hospital strategies that seek to improve quality, while reducing or containing costs. The aim of this study is to identify hospitals that have implemented such strategies and determine factors influencing the implementation.

**Methods:** We searched PubMed, EMBASE, Web of Science, Cochrane Library and EconLit for case studies on hospital-wide strategies aiming to increase quality and reduce costs. Additionally, grey literature databases, Google and selected websites were searched. We used inductive coding to identify factors relating to implementation of the strategies.

**Results:** The literature search identified 4198 papers, of which our included 17 papers describe 19 case studies from five countries, mostly from the United States. To accomplish their goals, hospitals use different management strategies, such as continuous quality improvement (CQI), clinical pathways, Lean, Six Sigma and value-based healthcare (VBHC). Reported effects on both quality and costs are predominantly positive. Factors identified to be relevant for implementation were categorized in eleven themes: (1) strategy, (2) leadership, (3) engagement, (4) reorganization, (5) finances, (6) data and information technology (IT), (7) projects, (8) support, (9) skill development, (10) culture, and (11) communication. Recurring barriers for implementation are a lack of physician engagement, insufficient financial support, and poor data collection.

**Conclusion:** Hospital strategies that explicitly aim to provide high quality care at low costs may be a promising option to bend the cost curve while improving quality. We found a limited amount of studies, and varying contexts across case studies. This underlines the importance of integrated evaluation research. When implementing a quality enhancing, cost reducing strategy, we recommend considering eleven conditions for successful implementation that we were able to derive from the literature.

## Background

Hospitals have grown into large and increasingly complex organizations.^1^ Fragmented and inefficient payment systems may incentivize supplier-induced demand, resulting in provision of services that are not strictly necessary or provide low-value to patients.^2^ As healthcare costs are becoming increasingly constraint, governments and third-parties aim to bend the cost curve. A triple aim perspective may require alignment of government and hospital strategies to increase quality while lowering costs.^3^ However, payer-initiated cost-containment policies may prove to be ineffective or harmful in a complex, adaptive hospital system.^4^ A promising option to bend the cost curve is a hospital-initiated strategy to increase quality while lowering the costs.

Research on strategies that focus on hospital quality improvement is abundant, as well as research focusing on strategies for hospital cost containment. Systematic reviews on quality improvement in healthcare demonstrated that evaluations often focus on either an assessment of cost-effectiveness or on quality improvement.^5,6^ Cost-control studies at the hospital level concentrate on global budgeting and other payment reforms.^4^ However, improving quality may be costly, while reducing costs may affect quality of care negatively, stressing the importance of an integrated approach.^7^ In this study, we focus on hospitals that aim to reduce costs through process quality improvements, rather than allocating financial resources to increase product quality. To our knowledge, no studies have explored which hospitals use integrated strategies aimed at quality improvements to reduce costs and factors associated with successful implementation.

 We collect case studies of integrated hospital strategies to improve quality and contain costs. The aim of this study is to identify hospitals that have implemented such strategies and identify important factors in implementation. The research questions are:

- Which hospitals have adopted a hospital-wide strategy aimed at improving quality and thus reducing costs?
- Which factors play a role in adopting these hospital-wide strategies?

Our aim is to identify hospitals that have developed and implemented such organization-wide strategies and, subsequently, analyze which factors have played a key role in this process.

 The paper is structured as follows. First, background information is given on hospital management theories and strategies to improve efficiency. Second, we elaborate our scoping review methodology. Third, results are presented, and important factors in implementation are defined. Last, the results are discussed, followed by a conclusion.

###  Hospital Management Trends

In their search for efficiency, hospitals have experimented with strategies from general management literature and have tailored such strategies for their own use. The management trends described below mostly focus on improving healthcare quality processes opposed to improving product quality: the former is associated with cost reductions, while the latter may increase costs.^8,9^

The balanced scorecard method was developed in industry in 1992, and later applied to healthcare organizations.^10^ A balanced scorecard strategy requires measuring and monitoring organizational goals, such as cost reductions or quality improvements.^11^ Total quality management (TQM), inspired by the successes of the Japanese industry sector, gave rise to a number of process oriented optimization strategies, which were first applied to hospitals in the 1990s.^12^ As a management strategy, TQM focuses on across-the-board quality improvements, which consequentially should reduce costs. Translation of TQM to the healthcare sector gave rise to certain related approaches, such as continuous quality improvement (CQI) programs, which were often heterogeneous projects focusing on quality improvements in wards and hospital departments^13^; and, Clinical Pathways methods - standardizing and streamlining patient pathways to increase quality while reducing waiting time, errors and costs^14^ - promoted interdepartmental cooperation and redefinition of workflows.^15^ Integrated care optimizes patient pathways across organizations, coordinating efforts of hospitals, primary care and long-term care in networks or integrated organizations.^16^ The Toyota Production System (TPS), originating in the Toyota company in Japan, incorporates elements of quality improvement and waste reduction.^17^ The TPS was adopted by manufacturing and service sectors as Lean management.^18^ Lean management focuses on reducing waste and inefficiencies in the production process.^19^ The Motorola Company translated Lean thinking into Six Sigma, a continuous improvement cycle aiming to reduce variation in the production process,^20^ resulting in less errors.^21^

In 2006 Porter and Teisberg developed value-based healthcare (VBHC) as a method to optimize outcomes.^22^ The aim of VBHC is to improve value of care, where value is defined as quality divided by costs.^23^ VBHC incorporates clinical pathways, integrated care, CQI and process optimization, and was specifically designed for healthcare.^24^ Recently, patient centered care was developed to counterbalance the many strategies that seek for standardization and rationality. They define value of care from a patient perspective.^25^ Empowering patients, eg, through Choosing Wisely campaigns, shared-decision making initiatives, both with the intention to improve patient outcomes and lower costs are branches of this patient-centered approach.^26^ In extension, person-centered care incorporates personal values beyond (clinical) care outcomes.^27^

## Methods

We conducted a scoping review to identify hospital strategies that aim to reduce costs and improve quality. Scoping reviews have previously been found to be effective in capturing a range of literature on a topic, allowing it to be summarized and compared.^28^ Compared to systematic review methods, a scoping review is suitable for mapping concepts, rather than answering specific questions on effectiveness or appropriateness of interventions.^29^ We conducted the scoping review according to the steps by Arksey and O’Malley^30^: (1) identifying the research question, (2) identifying relevant studies, (3) study selection, (4) charting the data and, finally, (5) collating, summarizing and reporting the results.

The following online literature databases were searched from inception until August 9, 2019: PubMed, EMBASE, Web of Science, Cochrane Library and EconLit. We used a combination of the following keywords (and synonyms): hospital, academic medical center, clinic; quality improvement, cost control, efficiency; strategy, organizational change; case study. The full search lay-out for PubMed is listed in Supplementary file 1. Other database searches were constructed using similar terms. In addition, grey literature was searched according to the protocol developed by Godin et al,^31^ consisting of 5 steps: (1) Grey literature databases: Open Grey, BASE, OAlster, WHOLIS (Supplementary file 2); (2) Custom google search, screening 1000 results (Supplementary file 2); (3) Hand-searching relevant websites (Supplementary file 2); (4) Consulting experts in the field; and (5) Screening reference lists using forward and backward snowballing procedures.

###  Study Selection

We explicitly sought to identify hospitals aiming to both increase quality and reduce costs with an organization-wide strategy. Therefore, case studies that study single departments were excluded, as well as hospital-wide strategies that focus on quality improvement exclusively, or hospital-wide strategies that only focus on cost containment. Moreover, we excluded strategies initiated by payers or integrated organizations, as our scope is limited to intra-organizational strategies. All inclusion and exclusion criteria are listed in Table 1.

**Table 1:** Inclusion and Exclusion Criteria

**Inclusion**	**Exclusion**
Strategy aims to increase quality and reduce costs	Quality improvement strategy only, cost containment strategy only
Strategy is implemented hospital-wide	Interventions focusing on single patient groups, wards or departments
Strategy is initiated by a hospital	Strategies initiated by payers or integrated organizations (including ACOs and partnerships)
Hospital is situated in an OECD member country	Hospitals outside OECD countries
English language	Not available in English language

Abbreviations: OECD, Organisation for Economic Co-operation and Development; ACOs, Accountable Care Organizations.

 All articles were screened on title and abstract by two researchers (EW and AH). Upon doubt, a third researcher was consulted (NS). Relevant articles (n = 89) were independently screened full-text by two researchers (EW, AH or NS) and discussed until agreement was reached.

 We extracted a short description of each case study, the institution name, year of implementation, country, type of strategy, and effects on quality and costs. Next, the articles were coded inductively by one researcher. Relevant factors related to barriers and facilitators in implementation were highlighted and extracted. The factors were inductively categorized into themes using open coding by one researcher based on similarity. Code groups and categorizations were discussed in detail by a team of three researchers. Any disagreements were recategorized based on consensus. All results are summarized using narrative synthesis.

We did not conduct a critical appraisal of included studies, as our primary objective was to identify case studies on this topic. Results are reported according to the Preferred Reporting Items for Systematic reviews and Meta-Analyses extension for scoping reviews (PRISMA-ScR) guidelines.^32^

## Results

In total, 4198 records were found after duplication removal. After title, abstract and full-text screening, 17 articles were included. One study reported three case studies, bringing the total to 19. The selection process according to the PRISMA statement is illustrated in Figure.^32^

**Figure:**
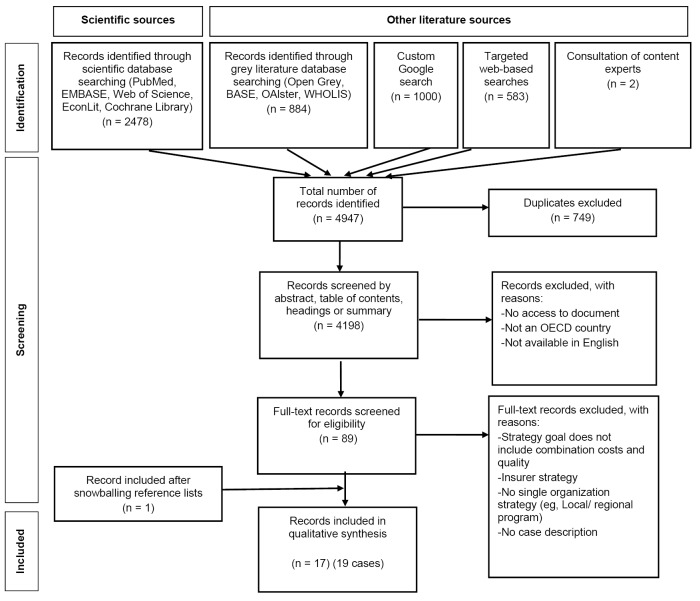
PRISMA Flow Diagram of the Literature Search. Abbreviations: PRISMA, Preferred Reporting Items for Systematic Reviews and Meta-Analysis; OECD, Organisation for Economic Co-operation and Development.

###  Study Characteristics

Table 2 reports case study characteristics for all 19 cases. Case studies were found from five countries, of which the United States was most predominant (n = 11). VBHC (n = 7) was the most prevalent management strategy, followed by Lean (n = 4) and Six Sigma (n = 2). Three case studies combine different strategies, such as VBHC and clinical pathways. In all but one case, new strategies were internally developed and the theoretical concepts from the literature were adjusted to its own needs; only the Academic Medical Center Groningen in the Netherlands ‘copied’ the existing hospital strategy of the much smaller Red Cross Hospital in Beverwijk as a blueprint for implementation. Earlier cases more often used CQI-based strategies, chronologically followed by TPS, Lean and Six Sigma strategies, while VBHC-strategies were more popular in more recent cases.

**Table 2:** Summary of Case Study Details

**Case Study**	**Author(s)**	**Country**	**Year**	**Type of Management Strategy Used**	**Reported Effect on Costs**	**Reported Effects on Quality**
University of Massachusetts Medical Center	Strongwater, 1996	USA	1989	CQI	Shorter length of stay, fewer diagnostic studies and reduction of pharmaceutical use. Approximately $6 million saved over 2 years.	Improvement of clinical outcomes.
Duke Children's Hospital	Meliones, 2000	USA	1996	Balanced Scorecard	Lower cost per case, net margin increased to $ 4 million.	Length of stay declined from 7.9 days in 1996 to 6.1 days in 2000, readmission rate dropped from 7% to 3%, customer satisfaction rates increased 18%.
Rady Children’s Hospital of San Diego	Reynolds and Roble, 2006	USA	1997	Clinical Pathways	50% decrease in variable costs per case.	Mortality and complication rates reduced by over 30%.
Banner Health Network (I)	Kirkman-Liff, 2004	USA	2001	Integrated care	Not mentioned.	Not mentioned.
Red Cross Hospital	Van den Heuvel et al, 2006	The Netherlands	2001	Six Sigma	€1.4 million savings in 2004.	Reduced waiting times and shorter length of stay.
Virginia Mason	Miller, 2010	USA	2002	TPS	$12 million savings annually.	Nurses have more time per patient, reduced waiting times.
Medical University of South Carolina	Rees, 2014	USA	2006	VBHC	17% reduction in costs per case.	11% reduction in length of stay.
Lawrence and Memorial Hospital	Birk, 2010	USA	2006	TPS	Not mentioned.	Lower length of stay, increased patient satisfaction, less complications, lower personnel injuries.
University Medical Center Groningen	Niemeijer et al, 2012	The Netherlands	2007	Lean and Six Sigma	14% to 30% reduction in diagnostic testing, translating to 10% reduction in cost per treatment; estimated €15 million savings for all projects combined.	Lower length of stay, reduction in unnecessary admissions.
Banner Health Network (II)	Kuhn and Lehn, 2015	USA	2011	Integrated care	$19; $15; $29 million savings in 2011, 2012, 2013.	Average length of stay reduced by 14.4% and avoidable hospital readmissions reduced by 6%.
Azienda Ospedaliera Universitaria Senese	Barnabé et al, 2019	Italy	2012	Lean	€5 417 395 in savings.	Reduced waiting times for patients.
Health First Florida	Blanchard and Rudin, 2015	USA	2012	Lean	Not mentioned.	Waiting times were reduced: ED times decreased 37%.
Royal Bolton Hospital	Jabbal and Lewis, 2018	UK	2012	Lean/VBHC/ integrated care	Improved financial position.	Waiting times decreased.
University Utah Healthcare	Lee et al, 2016	USA	2012	VBHC	7%-11% cost reductions in 2 projects.	Higher quality on composite quality index.
Sahlgrenska University Hospital	Nilsson et al, 2017	Sweden	2013	VBHC	Not mentioned.	Increased patient registration in national quality registries, reduced length of stay after readmission.
Bernhoven	Van Leersum et al, 2019	The Netherlands	2013	VBHC/ patient-centeredness/ integrated care	16% lower DRG claims after three years.	Patient satisfaction scores have gone up from 77% recommending the hospital to others in 2014, to 93% in 2018.
NYU Langone Health	Chatfield et al, 2019	USA	2014	VBHC	Costs have been reduced by 7.7% (in comparison to expectation), translates to approximately $53.9 million.	Lower length of stay (0.25% per month), high quality score.
Royal Free London Group	Jabbal and Lewis, 2018	UK	2017	VBHC/clinical pathways	Not mentioned.	Not mentioned.
Bradford Teaching Hospitals	Jabbal and Lewis, 2018	UK	2017	CQI	Lower income for the hospital due to reduced inpatient activity.	Lower length of stay.

Abbreviations: CQI, continuous quality improvement; TPS, Toyota Production System; VBHC, value-based healthcare; DRG, diagnosis-related group; ED, emergency department.

###  Reported Effects on Costs

 Most papers reported the annual (estimated) amount of savings in local currency. Lower costs per case were reported in four papers, while lower volumes were reported in two papers. Five papers did not report costs. The size of the effect differs greatly between cases, from relatively small savings of $50 000 to savings as large as $50 million. As cost savings in absolute numbers depend on the size of the hospital, reported savings in percentages of revenue display smaller ranges, between 7% and 17%. Only NYU Langone Health and University Utah Healthcare report reductions of -7.7% in adjusted variable costs and -11% in mean direct costs after one year, respectively.

###  Reported Effects on Quality

 Seventeen of 19 cases report effects on one or more quality parameters, most often length of stay (n = 9) and waiting times (n = 5). As clinical outcomes, avoidable readmissions (n = 3) and complications (n = 2) were mentioned, as well as scores on composite quality indices (n = 3). Patient satisfaction was mentioned in 3 papers and personnel outcomes were mentioned in 2 papers. In most cases, quality improvement was reported, although the strength of evidence is low. Strength of evidence on quality effects ranged from anecdotal (n = 10), to trend-based (n = 7), to quasi-experimental (n = 2).

###  Themes in Implementation of Strategies 

In total, 265 barriers and facilitators were extracted from the 19 cases. These were categorized inductively into 11 major themes: strategy, leadership, finances, engagement, projects, culture, support, reorganization, data collection, skill development, and communication. Table 3 provides an overview of the distribution of barriers and facilitators across themes and cases.

**Table 3:** Overview of Facilitators and Barriers

**Theme**	**Facilitators**	**Barriers**
Strategy	A detailed and clear strategy Establishing conservative, attainable goals Strategic approach to implementation Bottom-up strategy development Organic strategy development across the organization Flexible change process and learn from failures Structured plan Commitment to the strategy Setting key performance metrics Continuous improvement Focus on value Aligning internal goals with external accrediting agencies and financial imperatives	Emphasis on administrative aspects Focus exclusively on cost reduction Lack of a standard approach
Leadership	Leaders actively motivate change Appointment of champions Joint clinical and operational leadership Top management support	Power consolidation for doctors and managers Resistance to responsibility redefinition
Engagement	Shared problem ownership Physician feedback to management Alignment medical and nursing staff Engaging external stakeholders Engagement on all organizational levels Involvement and feedback to senior leaders Aligning goals for administrators and physicians Bottom-up implementation Sense of accountability Interdisciplinary groups	Lack of (visible) results Lack of physician involvement and commitment Lack of staff involvement Lack of patient involvement Poor accountability to outcomes
(Re-) organization	Reorganization to align organizational structure with strategy Organization-wide implementation ensures standardized methods Redefining roles Developing new protocols Facilitating a structure for communication between management and staff	Decentralised organization Uncoordinated implementation at different organizational levels Lack of coordination with external stakeholders
Finances	Financial incentives for physicians Financial sense of urgency for change Hospital-payer collaboration External grant support Initial investments Shared savings for physicians and departments	No staff reimbursement for involvement Difficult negotiations for distributing cost savings Complex financial structures Cost savings are demonstrated in the long term
Data/IT	Data-driven approach Using data feedback for accountability and improvement Creating scientific evidence Investments in measurement instruments Adequate IT infrastructure Transparent and timely use of data Using data for benchmarking with other organizations	Data paralysis: continually seeking new data without making progress No clear indicators to demonstrate improvement Incomplete data and reporting Inadequate data collection Poor measurement system
Projects	Project selection consistent with strategy Pilot projects to improve the strategy Rapid-cycle improvement Limited scope of projects Sustained focus on projects Shared responsibility for managers and physicians in project groups Incentives for physicians to initiate projects Predictable and structured approach of the project	Interventions beyond the scope of champions and department
Support	Formation of a support department for continuous improvement Project staff to support physicians External expertise for training and project management Frequent meetings with support staff	Resentment towards external consultants Inadequate collaboration between patient care units and support areas Implementation mostly dependent on external capacity
Skill development	Investment in human resources Staff training in new (IT) structure Education of physician leaders Learning from previous quality improvement efforts	Staff and management lacking competencies Insufficient training for new information system Lack of qualified personnel
Culture	Informal implementation initiatives Celebrating success Cultivating a sense of humour Positive peer pressure to improve Cultural change program Promoting cultural change through projects Aligning administrators and physicians (instead of “us versus them”)	Resistance to change Lack of awareness for improvement opportunities New routines require effort
Communication	Distributing lessons learned across the organization Sharing successes Techniques to improve communication between management and physicians Sense of humour in discussions Frequent evaluations and feedback	Excessive focus on details in the early stages of the program

Abbreviation: IT, information technology.

Overall, data and information technology (IT), engagement and strategy are the most addressed themes, whereas communication, reorganization and skill development are the least frequently addressed themes. Facilitators are reported more often than barriers (Supplementary file 3). We discuss individual themes in detail below.

###  Strategy

We identified several important factors related to the strategy. First of all, the strategy should be clear, credible and easy to understand.^33-35^ It should be detailed and specific, so all stakeholders involved should know their tasks, roles and activities.^36^ Furthermore, the strategic aims should be ambitious, eg, to become the health leader in certain areas.^37^ However, short-term goals should be conservative to build trust and momentum; changing everything at once could lead to failure.^37^ Organic strategies could be more suited for implementation across the organization,^34^ as flexibility in the strategy could help in experimenting, learning from errors and improving the strategy along the way.^33,37^ Consistent leadership commitment to the strategy is important throughout the process.^34^

Moreover, a bottom-up strategy involving all employees to think along, could assist implementation and encourage strategic thinking at all levels of the organization.^33,34,38^ Broad support may be easier to obtain when the strategy focuses on value creation instead of cost reductions.^39^ Conversely, perceived emphasis on cost containment and administrative aspects of the program, potentially reducing resources for patient care, was found to be a barrier in implementation.^33^

###  Leadership

Overall, case studies indicate leadership should be supportive and aware of existing power dynamics to successfully implement the program. Successful implementation requires strong leaders at top positions, such as the Chief Financial Officer and the Chief Medical Officer.^35,40-43^ At the operational level, the appointment of champions, such as key surgeons, is recommended to increase support.^37^ Resistance to shifts in power could, however, present a barrier for implementation for doctors and managers.^37^ For example, managers at the University Medical Center Groningen in the Netherlands were reluctant to allow interference in their departments.^42^ Generally, reduced responsibility is met with opposition.^35^ A predictable and structured approach helps to overcome resistance to change.^33,37^

Urgency for improvement is not always perceived, as staff is accustomed to the existing processes.^35^ By showing support and commitment, leadership can signal that the strategic change is an institutional priority.^34,39,42,44^ Joint leadership, shared between the physician and the manager, improves commitment and accountability and helps to build consensus.^37,39^ Staff should also be empowered to lead improvements.^34^ Proactive frontline managers are required to initiate change to improve performance.^34,35^

###  Engagement 

The majority of case studies demonstrate that engagement of physicians and other relevant stakeholders is an important element of successful implementation. Physician involvement, especially of senior leaders, is critical for a successful strategy.^33,43,45,46^ Administrators should also be highly involved.^37,38^ This may take a great deal of persuasion by senior management, as doctors and managers may view the strategic change as a shift in their power base.^37^ A sense of accountability of hospital employees and medical staff around the aims of the strategy catalyzes engagement.^34,45^ Physician engagement could improve ownership of the new process,^35,45^ and foster learning and growth.^43^ In addition, alignment of nurses and clinical staff could improve implementation and outcomes.^33^

Engagement should also be broadened towards relevant stakeholders outside the hospital.^34^ Engaging hospital partners has been key for success.^44^ For example, active engagement of primary care physicians could help to substitute care to the primary setting.^38,44^ Cooperation with other hospitals may be warranted to help make improvements.^46^ Engagement requires good communication of critical information to relevant stakeholders.^44^

A number of barriers for implementation have also been identified. A top-down approach has led to aversion towards the program^33^ and reduced effectivities of initiatives.^41^ Furthermore, a lack of results negatively affects physicians’ engagement.^33,45^ Finally, patient engagement in the design of many improvement initiatives has been lacking, missing an opportunity to increase service value.^34^

###  (Re-)organization 

A new organizational strategy may require a restructured organization as well. An identified barrier in implementation is a lack of coordination between projects and departments.^37,42,44^ Reorganization could reduce organizational silos and improve collaboration.^38^ For example, the hospital structure may be reorganized according to patient needs.^38^ Reorganization may also require a redefinition of roles, for example from being a nurse to being part of a project team.^37^ While implementation takes place at the work floor, oversight should be directed centrally.^33,45^ A central body may therefore be required to address common problems in implementation in different departments.^42^

###  Finances 

Finances eminently act as both a facilitating and limiting precondition. Foremost, financial difficulties create a sense of urgency, which helps to implement change.^34,37,45^ However, initial investments, or ‘seed money,’ may be necessary to support implementation,^33,34,38^ albeit only minor financial investments in some cases.^40^ Collaboration between hospitals and payers is often required to realize cost savings and quality improvements. ^33,38^ This may require contract innovation, such as shared savings,^38,39^ and thus innovative payers.^40^ A degree of mutual trust is required, as payers may demand additional cutbacks in the future.^40^

Due to the complex financial structure of large hospitals, certain interventions may be met with resistance, as projects may cross several revenue streams and internal budgets^42^ In order to increase support for implementation, physicians should be compensated financially.^40^ Insufficient compensation for increased work and time input has a negative effect on support for the program.^33^ It may be challenging to negotiate a fair distribution of potential cost savings among stakeholders.^40^ Furthermore, cost savings typically have been observed after a longer period of time, making it difficult to demonstrate short-term results.^39^

###  Data and Information Technology 

Frequently addressed, use of data and analysis is an essential factor. First, data can be used by leaders to establish priorities and identify gaps in performance and identify potential cost savings.^39,45^ Second, providing data feedback to physicians may convince them of the need to change.^33,35,39,40^ Physicians generally respond well to data.^46^ Meaningful data and feedback stimulates incentives to improve, increasing performance accountability.^37,38^ A data-driven approach could counterbalance subjective and intuitive viewpoints.^36^ Third, data is required to show improvements, which helps in building support for further implementation.^35,44^ This requires accurate measurement of baseline performance on important dimensions of care.^33^ Fourth, advanced IT helps to collect valuable information about patients, and monitoring may help improve patient safety and quality.^33,44^ Benchmarking to other providers and organizations helps identify outliers and deviations.^39,44^ Indicators should be universally applicable, such as inappropriate hospital stays.^42^ In order to be effective, data generation should be timely and useable.^34,35,47^ Adequate resources must be liberated for data collection and synthesis, for example investments in IT infrastructure^33,44^ Electronic health records are an important tool in improving patient safety, clinical excellence and operating efficiency.^44^ A customized information system may be necessary to support strategy implementation.^37^ This requires, however, staff to be able to interpret measurements and statistics.^37^ Several case studies indicate inadequate data collection as an important barrier.^42-44^ Data collection is complicated by a lack of clear measures and indicators for effective improvement strategies.^34,36^

###  Projects 

Organization-wide strategies often translate to several operational projects, targeting different departments. Strategic change requires employees to experience problems themselves, and design their own solutions.^42^ Therefore, physicians should be persuaded to create innovative ideas for projects.^33^ For example, case management to address chronic care of elderly may be employed as a project^33,44^ Projects that were beyond the scope of the appointed “champions,” were found to be less successful.^42^ Dual management of projects could help improve success of the project from both a clinical and managerial perspective.^37,39^ An interdisciplinary group helps to make change stick.^45^

Successful projects are reassuring and can reduce resistance to change.^33^ Therefore, carefully selected pilot projects may be important to learn and improve support.^37,43^ Furthermore, a sustained focus is necessary, as it generally takes some time before the projects show results.^34^ Rapid cycle improvements, meeting at high frequency, could help projects to swiftly effect change.^45^

###  Support 

Along the implementation process, project support is key.^47^ Support personnel is needed to help preoccupied faculty staff, which may require formation of a new project support team or department^33,39,47^ It is especially valuable to have project staff to support project teams, reducing meeting time and interruptions in daily activities.^33,39^ Interaction with project staff should be encouraged.^33,47^ Internal or external consultants may be employed to support project teams.^33,34,38,42,45^ External support capacity may, however, lead to resistance within internal staff, who have invested in the program^33^ and a loss of independence.^42^ Moreover, a gap may form between patient care and support units, hampering collaboration.^45^

###  Skill Development

Investments in human resources, as well as information infrastructures, are necessary for process improvement.^33,43^ Barriers arise when competencies and skills are insufficiently present.^33,41,44,46^ Therefore, skills of internal project managers and clinical leaders may need to be built.^33,44^ Training of professionals fosters learning and growth.^34,43^ Learning from past mistakes is essential in producing sustained results.^45^ When new data tools are used, staff needs to be trained and reminded in data processing tasks.^35^ In some cases, staff was unfamiliar with a new information system, delaying the implementation.^35,46^

###  Culture 

Culture change may be necessary to implement the strategy.^38^ It is important to create a culture where leaders listen to employees, where physicians and managers move past the “us versus them” mindset.^37,45^ Strategic implementation could spur spontaneous improvement efforts across the hospital.^33^ Success breeds success; successful projects help in widespread implementation.^37^ Therefore, successes should be celebrated.^37^ Positive recognition of successful leaders may induce positive peer pressure to improve.^33,45^ Chartering projects can help involve more physicians and promote culture change.^39^ Finally, a sense of humor may be helpful in the transformation.^37^

###  Communication 

Although factors connected to communication were not addressed frequently, it is strongly related to other important themes, such as strategy, engagement and leadership. Knowledge learned in the change process needs to be efficiently distributed across the organization.^47^ Communication with physicians requires subtle communication techniques, and feedback and evaluations help to keep the implementation process on track.^37^ Different media may be used, for example: emails, newsletters, websites, conference calls and educational conferences.^47^ Successes should be shared across the organization, in order to learn from each other’s’ experiences and build morale.^37^ Hospital successes also need to be showcased externally to raise the recognition of the organization.^34^ A potential barrier in communication is an increased focus on semantics, while the main focus should be on patients and staff.^37^

## Discussion

 In this review, we identified 19 case studies of hospitals that have implemented a strategy to improve quality while reducing costs. The limited number of studies demonstrate that these types of strategies are not commonplace. Since all three major types of health systems – national health service, social health insurance and private health insurance – are represented, the implementation of such a hospital strategy may not necessarily depend on health system type. The most dominant management theories on which the strategies are based were VBHC, Lean and Six Sigma, followed by TQM. The trend in hospital strategies over time broadly follows the evolution in management theories, from TQM to Lean and Six Sigma to VBHC. Reported effects on costs and quality were predominantly positive.

 We identified eleven themes that were relevant in implementation of the strategies: (1) strategy, (2) leadership, (3) engagement, (4) reorganization, (5) finances, (6) data and IT, (7) projects, (8) support, (9) skill development, (10) culture, and (11) communication. Barriers and facilitators of a successful hospital strategy reported in the case studies were categorized across these eleven themes. However, the themes are interrelated and interdependent, which implicates that strategies targeting single themes may have unanticipated effects in other themes. Classifications across fewer themes may reduce overlap, but also result in a more abstract representation of the results.

The themes closely follow existing literature on organizational change.^48-50^ Kotter’s eight-step model for change^49^ emphasizes the importance of “defrosting” the status quo with vision and strong leadership, before introducing structural changes and anchoring them in organizational culture. A perceived advantage for all stakeholders over the current situation is critical in adopting innovations.^50^

A well-formulated strategy was often experienced as a facilitator in implementation, as it provides clarity and guidance. This has also previously been linked to improved performance.^51^ The value of clinical leadership has also been demonstrated.^52^ This may require additional training of physicians.^34,43,44,52^ Continuous support, involvement, and belief in the new management style from top management and department managers is of utmost importance for successful adoption and implementation.^44,36,53,54^ When management is involved during implementation, staff will be less likely to resist changes. Improvement initiatives that do not have sufficient management commitment have low chances of success.^55-57^

Top-down leadership might, however, facilitate bottom-up engagement. Engagement of physicians and other stakeholders can be increased by project ownership, bottom-up project design and implementation, and dual management. Almost all included strategies are designed in-house. This illustrates the importance of a bottom-up approach as well as the challenge to spread successful strategies to other organizations.^58,59^ Strategies may be more effective when designed locally and through bottom-up processes.^60^ The impetus for improvement initiatives was internally driven in most cases.^34^ Pilot projects could help to increase focus and attention, creating momentum through sharing of successes. This requires clear communication and strong project support. Projects were often paired with a redefinition of roles, which required reconsideration of the current organizational design. A central body of support helped to implement projects across organizational siloes. While some cases rely on external consultants, most cases employ internal dedicated teams to support projects. Accountability for the results of the projects is important and requires extensive data creation and sharing.

Most strategies needed initial investments, seed money or external grants.^33,34,38^ Initial investments were necessary for training of personnel^36,44^ and adequate investment in IT infrastructure.^33,39,44^ When investments were not sufficient, effectiveness of the implementation of quality improvement initiatives was reduced.^55^ These initial investments may pose a problem for hospitals in a fiscally constrained environment. On the other hand, financial problems have acted as facilitators in strategy implementation, creating a sense of urgency.^34,37,45^ Funding initial investments may require innovative funding solutions with payers, of which shared savings contracts were named most often. However, this requires trust in payers not fully capitalizing the strategy gains in the future.

The generation of timely and accurate data is crucial to the success of implementation in most case studies to serve a number of aims: identifying opportunities, convincing physicians, generating evidence, monitoring and feedback. A lack of measurement, both of health outcomes and costs of healthcare or care processes, reduces physician accountability and commitment.^53-55^ In modern healthcare systems, many care- and health-related measurements are stored in a patient’s electronic health record. However, it is imperative that valuable information stored in the electronic health record can be exchanged easily and reliably between care providers.^53-55,61^

It is difficult to attribute specific strategies to themes, given the limited amount of studies we found. The overview of barriers and facilitators across case studies provided in Supplementary file 3 shows that themes are distributed randomly across case studies. Themes such as engagement and data and IT were mentioned in the majority of case studies. The aspect of culture was mostly mentioned in hospitals using VBHC, CQI strategies and balanced score card strategies.^33,37,38,41^ Furthermore, no patterns can be discerned in influencing factors over time (ie, early 90s until present), while the terminology in strategies did change. Overall, we do not see any clear cross-links between type of strategy and themes, which may indicate the similarity in this variety of strategies in terms of development and implementation.

###  Limitations

 This research experiences several limitations. Selection bias relates to preconditions present at the included hospitals, that may have given them a competitive advantage in implementing strategic change. This implicates that broad adoption of hospitals strategies may produce less favorable results. Risk of publication bias is high, as hospitals that failed in implementing a strategy aimed at high quality and low costs are less likely to publish their results as a case study. This biases the results towards successful implementation, as demonstrated by the overwhelmingly positive effects reported. Furthermore, the strength of evidence was generally low, and the context of hospitals varied. These findings are therefore not generalizable across contexts. As the primary objective of this study was to identify relevant case studies, this did not affect the results. Because case studies require extensive information from within the hospital, case studies are often written by hospital personnel, risking reporting bias, ie, selectively reporting the results that were positive. Only 7 of 19 case studies were written by independent authors. These biases imply that caution should be taken in generalizing the findings to other hospitals in different contexts.

###  Recommendations

 Great variety was found in strategies for hospitals. The selected cases also portray a wide array of sizes, geographical locations, histories and services. This is noteworthy, as all strategies have the same aim – higher quality and lower costs – but show varying improvement processes. Based on our results, the specific type of strategy is secondary to the eleven themes that were found conditional for successful implementation. Although context may differ, these themes could serve as a checklist for hospital management, medical personnel or staff looking to improve their organization in terms of quality and costs. The eleven themes are interdependent: strategy elements targeting bottom-up engagement, for example, may impact top-down leadership and vice versa. Hospital managers should therefore aim to balance efforts across themes and detect potential conflicts. The added value for hospital management has been demonstrated throughout this study.

Studies evaluating the effect of hospital-wide strategies on both quality and costs are scarce.^7^ Considering the broad search conducted in this study, we have found relatively few cases. Strengthened by previous work into quality improvements aimed at reducing costs,^7^ we encourage researchers to conduct additional case studies of hospitals that have taken a leap at higher quality and low costs.

## Conclusion

 This scoping review presented 19 case studies of hospitals that have implemented an integral strategy to increase quality and reduce costs. This could be a promising policy option to bend the cost curve while improving quality of care. We identified eleven themes that hospitals should take into account upon implementation. When implementing a quality enhancing, cost reducing strategy, we recommend to base such strategy on eleven conditions for successful implementation we were able to derive from the literature.

## Ethical issues

 Not applicable.

## Competing interests

 Authors declare that they have no competing interests.

## Authors’ contributions

 All authors contributed to the design of the study. EW, NS, and AH were involved in the selection and synthesis process. EW and NS drafted the manuscript and all authors contributed to its revision substantially.

## Funding

 This work was supported by the Ministry of Health, Welfare and Sport, the Hague, the Netherlands.

## Supplementary files

Supplementary file 1. Search Lay-Out PubMed.

Supplementary file 2. Grey Literature Search.

Supplementary file 3. Overview of Barriers and Facilitators in 11 Themes From 19 Cases.
