# Supplementary material for: Hospitals Bending the Cost Curve With Increased Quality: A Scoping Review Into Integrated Hospital Strategies
Source: Int J Health Policy Manag. 2021 Dec 8;11(11):2381–91. doi: 10.34172/ijhpm.2021.168 (PMC9818083; doi:10.34172/ijhpm.2021.168)
Supplement: Supplementary file 1 — Search Lay-Out PubMed. [file ijhpm-11-2381-s001.pdf]

Article title: Hospitals Bending the Cost Curve With Increased Quality: A Scoping Review Into Integrated Hospital Strategies

Journal name: International Journal of Health Policy and Management (IJHPM)

Authors' information: Erik Wackers[1]*, Niek Stadhouders[1], Anthony Heil[1], Gert Westert[1], Simone van Dulmen[1], Patrick Jeurissen[1,2]

[1]Radboud University Medical Center, Radboud Institute for Health Sciences, IQ Healthcare, Nijmegen, The Netherlands.

[2]Ministry of Health, Welfare, and Sport, The Hague, The Netherlands.

(*Corresponding author: Erik.Wackers@radboudumc.nl)

Supplementary file 1. Search Lay-Out PubMed

Block 1: Population
(("Hospitals"[Mesh] OR "Academic Medical Centers"[Mesh]) OR (Hospital[tiab] OR Hospitals[tiab] OR Clinic[tiab] OR Clinics[tiab] OR (Health[tiab] AND (Organization*[tiab] OR Organisation*[tiab]))))
AND

Block 2: Intervention
(("Quality Improvement"[Mesh:NoExp] OR "Quality of Health Care"[Mesh:NoExp] OR Quality[tiab] OR health gain[tiab]) AND
("Cost Control"[Mesh] OR "Hospital Costs"[Mesh] OR "Health Expenditures"[Mesh:NoExp] OR Cost[tiab] OR Costs[tiab] OR Spending[tiab] OR Expenditure[tiab] OR Expenditures[tiab]) OR (value based healthcare[tiab] OR VBHC[tiab] OR triple aim[tiab] OR ((optimiz*[tiab] OR Maximiz*[tiab] OR optimis*[tiab] OR Maximis*[tiab]) AND (Value[tiab] OR Efficiency[tiab])) OR ((lean[tiab]) AND (philosophy[tiab] OR management[tiab] OR methodology[tiab] OR concept[tiab] OR concepts[tiab] OR process[tiab] OR processes[tiab] OR practice[tiab] OR practices[tiab] OR thinking[tiab] OR transformation[tiab] OR transformations[tiab] OR six sigma[tiab] OR production[tiab])) OR Kaizen[tiab] OR shared savings[tiab] OR population-based payments[tiab] OR population payments[tiab] OR p4p[tiab] OR pfp[tiab] OR pay-for-performance[tiab]))
AND

Block 3: Implementation
(("Program Evaluation"[Mesh] OR "Organizational Innovation"[Mesh]) OR (culture[tiab] OR strategy[tiab] OR strategies[tiab] OR improvement cycle[tiab] OR Program Evaluation[tiab] OR Program Effectiveness[tiab] OR Organizational Change*[tiab] OR Organisational Change*[tiab] OR Organizational Innovation*[tiab] OR Organisational Innovation*[tiab] OR preventive program*[tiab])
AND

Block 4: Study type
(("Organizational Case Studies"[Mesh] OR Case study[tiab] OR case studies[tiab]) OR review [ti] OR integrated pilot[tiab]))
