# Supplementary material for: Hospitals Bending the Cost Curve With Increased Quality: A Scoping Review Into Integrated Hospital Strategies
Source: Int J Health Policy Manag. 2021 Dec 8;11(11):2381–91. doi: 10.34172/ijhpm.2021.168 (PMC9818083; doi:10.34172/ijhpm.2021.168)
Supplement: Supplementary file 2 — Grey Literature Search. [file ijhpm-11-2381-s002.pdf]

Article title: Hospitals Bending the Cost Curve With Increased Quality: A Scoping Review Into Integrated Hospital Strategies

Journal name: International Journal of Health Policy and Management (IJHPM)

Authors' information: Erik Wackers[1]*, Niek Stadhouders[1], Anthony Heil[1], Gert Westert[1], Simone van Dulmen[1], Patrick Jeurissen[1,2]

[1]Radboud University Medical Center, Radboud Institute for Health Sciences, IQ Healthcare, Nijmegen, The Netherlands.

[2]Ministry of Health, Welfare, and Sport, The Hague, The Netherlands.

(*Corresponding author: Erik.Wackers@radboudumc.nl)

Supplementary file 2. Grey Literature Search

Table S1: Grey Literature Databases (Date Searched: March 12, 2019)

**Database**	**Search term**	**Results**	**Available English/ Dutch**	**Full-text screening**
Open Grey	Hospital quality improvement	75	34	0
BASE	Hospital quality improvement cost containment strategy	458	374	1
OAlster	Hospital quality improvement cost containment	146	60 English<br>19 Dutch	4
WHOLIS	Hospital quality improvement	2	2	0
Cumulative		884		

Table S2: Custom Google Search (Date Searched: April 4, 2019)

Nr	Search	Results	Screened	Full-text screening	Cumulative
1	Hospital AND Quality AND Cost AND Strategy	184.000.000	100	6	6
2	Hospital AND Quality AND Cost AND Case study	179.000.000	100	4	10
3	Hospital AND Quality improvement AND Cost savings OR Cost containment	6.510.000	100	3	13
4	Hospital AND Quality improvement program AND Case study	194.000.000	100	5	18
5	Hospital AND Quality improvement AND cost savings OR cost containment AND case study	199.000.000	100	1	19
6	Hospital AND Quality of care AND Cost reduction AND Case study OR Strategy	174.000.000	100	3	22
7	Hospital AND Quality improvement AND Health expenditure AND Case Study OR Strategy	58.700.000	100	0	22
8	Hospital AND Health gain AND Spending reduction AND Case Study	42.700.000	100	2	24
9	Hospital AND Quality improvement AND Cost control AND Case study	171.000.000	100	1	25
10	Hospital AND Quality improvement AND Cost efficiency AND Case study	163.000.000	100	0	25

Table S3: List of Websites for Grey Literature Search (Date Searched: August 15, 2019)

**Website**	**Search string**	**Hits**	**Full-text screening**
The Kings Fund	"hospital quality improvement cost reduction"	98	1
The Commonwealth Fund	"hospital quality improvement"	20	3
Harvard Business Review	"hospital quality improvement cost reduction"	28	0
American Hospital Association (AHA)	"hospital quality improvement cost reduction case study"	11038 (Screened first 50)	0
Boston Consultancy Group	"hospital quality improvement cost reduction"	27	1
Rand Corporation	"hospital quality improvement cost reduction case study"	45	1
Zorginstituut	"ziekenhuis kwaliteitsverbetering"	44	0
HC innovation group	"hospital quality improvement"	51	1
The Health Foundation	"hospital quality improvement cost reduction case study"	2704 (Screened first 50)	2
Care Quality Commission	"hospital quality improvement cost reduction case study"	9492 (Screened first 50)	2
OECD	"hospital quality improvement cost reduction case study"	5110 (Screened first 50	0
WHO	"hospital quality improvement cost reduction case study"	4157 (Screened first 50)	0
