# Supplementary material for: Hospitals Bending the Cost Curve With Increased Quality: A Scoping Review Into Integrated Hospital Strategies
Source: Int J Health Policy Manag. 2021 Dec 8;11(11):2381–91. doi: 10.34172/ijhpm.2021.168 (PMC9818083; doi:10.34172/ijhpm.2021.168)
Supplement: Supplementary file 3 — Overview of Barriers and Facilitators in 11 Themes From 19 Cases. [file ijhpm-11-2381-s003.pdf]

Article title: Hospitals Bending the Cost Curve With Increased Quality: A Scoping Review Into Integrated Hospital Strategies

Journal name: International Journal of Health Policy and Management (IJHPM)

Authors' information: Erik Wackers[1]*, Niek Stadhouders[1], Anthony Heil[1], Gert Westert[1], Simone van Dulmen[1], Patrick Jeurissen[1,2]

[1]Radboud University Medical Center, Radboud Institute for Health Sciences, IQ Healthcare, Nijmegen, The Netherlands.

[2]Ministry of Health, Welfare, and Sport, The Hague, The Netherlands.

(*Corresponding author: Erik.Wackers@radboudumc.nl)

Supplementary file 3. Overview of Barriers and Facilitators in 11 Themes From 19 Cases

Table S1: Overview of Barriers and Facilitators in 11 Themes from 19 Cases

		UMMC	DCH	BHN (I)	RCH-SD	RCH	VM	LMH	UMCG	MUSC	BHN (II)	HFF	UUH	SUH	RBH/BTH/RFLG	AOUS	BH	NYU-LH	Total
Strategy	F	8	8	2	.	1	.	.	.	2	3	1	.	.	5	.	1	1	32
	B	2	.	.	.	.	.	.	.	1	.	.	.	.	.	.	.	.	3
Leadership	F	2	3	.	1	.	.	.	2	.	1	4	.	1	4	1	1	2	22
	B	.	1	.	.	.	.	.	1	.	.	1	.	.	.	.	.	.	3
Engagement	F	5	2	1	.	.	.	2	.	5	5	2	.	.	5	3	2	.	32
	B	4	.	.	.	.	.	.	.	2	.	.	.	1	1	.	.	.	8
(Re-) organization	F	1	4	.	.	.	.	.	1	1	.	.	.	.	1	.	2	.	1
	B	.	.	.	.	.	.	.	2	.	1	.	.	.	.	.	.	.	3
Finances	F	3	4	.	4	.	.	.	.	2	.	.	.	.	3	.	3	2	21
	B	3	.	.	1	.	.	.	1	.	.	.	.	.	.	.	.	1	6
Data / IT	F	9	7	3	1	1	.	1	1	1	6	4	.	.	2	.	.	5	41
	B	1	.	.	.	1	.	.	1	1	.	.	.	.	1	3	.	.	8
Projects	F	4	4	.	.	.	.	.	3	1	1	.	.	.	1	1	.	1	16
	B	.	.	.	.	.	.	.	1	.	.	.	.	.	.	.	.	.	1
Support	F	8	.	2	.	.	.	.	1	1	.	.	.	.	1	.	1	2	16
	B	1	.	.	.	.	.	.	1	1	.	.	.	.	.	.	.	.	3
Skill development	F	1	.	1	.	.	.	.	.	2	1	1	.	.	1	2	.	.	9
	B	1	.	.	.	.	.	1	.	.	1	1	.	1	.	.	.	.	5
Culture	F	3	4	.	.	.	.	.	.	2	.	.	.	.	.	.	1	1	11
	B	1	1	.	.	.	.	.	.	.	.	1	.	1	.	.	.	.	4
Com-munication	F	.	5	3	.	.	.	.	.	.	.	.	.	.	1	.	.	.	9
	B	.	2	.	.	.	.	.	.	.	.	.	.	.	.	.	.	.	2

Abbreviations:UMMC=University of Massachusetts Medical Center; DCH=Duke Children's Hospital; BHN (I)=Banner Health Network (I); RCHSD=Rady Children's Hospital of San Diego; RCH=Red Cross Hospital; VM=Virginia Mason; LMH=Lawrence & Memorial Hospital; UMCG=University Medical Center Groningen; MUSC=Medical University of South Carolina; BHN (II)=Banner Health Network (II); HFF=Health First Florida; UUH=University Utah Healthcare; SUH=Sahlgrenska University Hospital; RBH=Royal Bolton Hospital; BTH=Bradford Teaching Hospitals; RFLG=Royal Free London Group; AOUS=Azienda Ospedaliera Universitaria Senese; BH= Bernhoven; NYU-LH=New York University Langone Health
